# Interleukin-1 in cardiac injury, repair, and remodeling: pathophysiologic and translational concepts

**DOI:** 10.15190/d.2015.33

**Published:** 2015-03-31

**Authors:** Nikolaos G. Frangogiannis

**Affiliations:** The Wilf Family Cardiovascular Research Institute, Department of Medicine (Cardiology), Albert Einstein College of Medicine, Bronx NY, USA

**Keywords:** Myocardial infarction, inflammation, cytokine, cardiac remodeling

## Abstract

In the infarcted myocardium, necrotic cardiomyocytes release danger signals activating an intense inflammatory reaction that serves to clear the wound from dead cells and matrix debris, but may also extend injury. A growing body of evidence suggests an important role for members of the Interleukin (IL)-1 family in injury, repair and remodeling of the infarcted heart. This review manuscript discusses the pathophysiologic functions of IL-1 in the infarcted and remodeling myocardium and its potential role as a therapeutic target in patients with myocardial infarction. Dead cardiomyocytes release IL-1a that may function as a crucial alarmin triggering the post-infarction inflammatory reaction. IL-1b is markedly upregulated in the infarcted myocardium; activation of the inflammasome in both cardiomyocytes and interstitial cells results in release of bioactive IL-1b in the infarcted area. Binding of IL-1 to the type 1 receptor triggers an inflammatory cascade, inducing recruitment of pro-inflammatory leukocytes and stimulating a matrix-degrading program in fibroblasts, while delaying myofibroblast conversion. IL-1 mediates dilative remodeling following infarction and may play a role in the pathogenesis of post-infarction heart failure. As the wound is cleared from dead cells and matrix debris, endogenous inhibitory signals suppress the IL-1 response resulting in repression of inflammation and resolution of the inflammatory infiltrate. Other members of the IL-1 family (such as IL-18 and IL-33) are also implicated in regulation of the inflammatory and reparative response following myocardial infarction. IL-18 may participate in pro-inflammatory signaling, whereas IL-33 may exert cytoprotective effects. Early clinical trials suggest that IL-1 blockade may be a promising therapeutic strategy for patients with myocardial infarction.

## 1. Introducion

Myocardial infarction triggers an intense inflammatory reaction that serves to clear the wound from dead cells and matrix debris and sets the stage for cardiac repair^1^. Necrotic cardiomyocytes release danger signals that activate innate immune pathways^2^, inducing secretion of pro-inflammatory cytokines and chemokines and stimulating adhesion molecule expression by endothelial cells^3,4^. Activation of adhesive interactions between leukocytes and endothelial cells results in intense infiltration of the infarct with neutrophils and mononuclear cells, which are predominantly localized in the infarct border zone. 20-30 years ago, a large body of experimental evidence, primarily derived from studies in large animal models of reperfused myocardial infarction suggested that inflammatory leukocytes may extend ischemic injury following myocardial infarction^5^. Broad anti-inflammatory strategies (such as corticosteroids) were tested; however, their wide range of effects on both inflammatory and reparative cells resulted (in some studies) in catastrophic consequences^6^. More selective strategies inhibiting specific adhesion molecules (such as leukocyte integrins or endothelial selectins) showed great promise in experimental models, markedly reducing the size of the infarct^7^. Unfortunately, despite the promising findings of the experimental investigations, small clinical trials targeting leukocyte integrins had disappointing results^7^. These failures greatly diminished enthusiasm regarding inflammatory targets in myocardial infarction. This is unfortunate, considering the important role of inflammatory mediators in adverse remodeling of the infarct heart and in the pathogenesis of heart failure that may not result solely from effects on cardiomyocyte survival. Thus, the quest for new inflammatory targets in myocardial infarction continues.

As the prototypical pro-inflammatory cytokine, interleukin (IL)-1 is involved in the pathogenesis of a wide range of inflammatory diseases^8,9^. IL-1 blockade is the standard of care for treatment of “autoinflammatory diseases”, a family of conditions characterized by dysfunction of monocytes/macrophages and recurrent bouts of debilitating inflammation^10^. Moreover, IL-1 neutralization therapy was beneficial in patients with rheumatoid arthritis, reducing symptoms and delaying the progression of joint destruction^11^. Emerging evidence suggests an important role for members of the IL-1 family in the pathogenesis of post-infarction cardiac remodeling and heart failure. This review manuscript discusses the role of IL-1 signaling in injury, repair and remodeling of the infarcted heart and its potential role as a therapeutic target.

## **2. ** The IL-1 family of cytokines

The IL-1 family is comprised of 7 agonist molecules (IL-1α, IL-1β, IL-18, IL-33, IL-36α, IL-36b and IL-36g), 3 receptor antagonists (Ra; IL-1Ra, IL-36Ra and IL-38) and the anti-inflammatory cytokine IL-37^12^. IL-1 family agonist molecules exhibit profound differences in their expression and regulation. Some members of the family, (such as IL-1α) are constitutively present as precursor proteins in most healthy cells and are released following injury, serving as typical alarmins. Other members (such as IL-1β and IL-18) are generally not expressed by healthy cells, but are synthesized as inactive precursors following stimulation and are processed to generate the active molecules. Generation of active IL-1β is primarily mediated through processing of the precursor protein by the intracellular cysteine protease caspase-1; however, caspase-1-independent mechanisms of IL-1β activation have also been described^13^. Activation of caspase-1 requires formation of a multi-component platform termed the “inflammasome”; one of the components of the inflammasome, Nucleotide-binding oligomerization domain-Like Receptor with a Pyrin domain 3 (NLRP3), plays an important role in generation of active IL-1β^14^.

**Figure 1:**
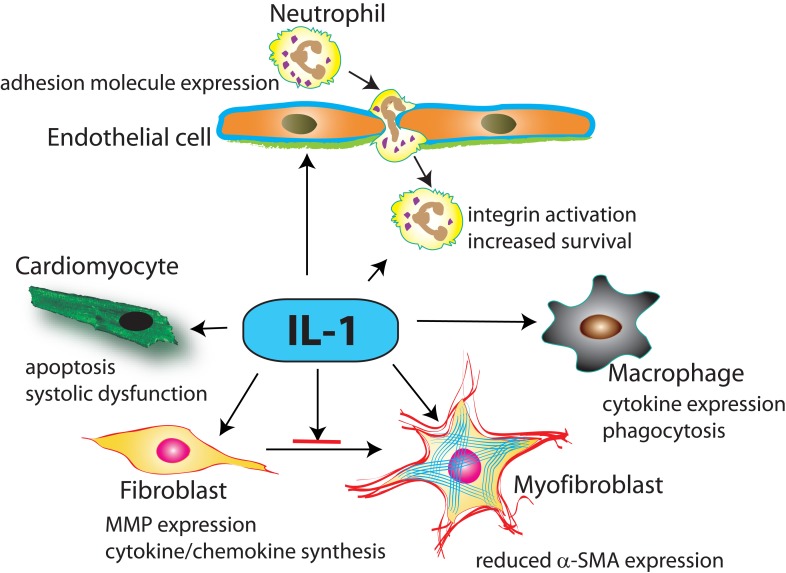
Figure 1. Regulation of IL-1 signaling by native inhibitors and decoy receptors IL-1α, or IL-1β binds to IL-1R1 and forms a complex with IL-1RAcP, recruiting myeloid differentiation protein 88 (MyD88) and triggering the signaling cascade that involves IRAK-1 and IRAK-4 activation **(A)**. The endogenous inhibitor IL-1Ra also binds to IL-1R1, but does not signal because it fails to form a complex with IL-1RAcP **(B)**. IL-1 binding to the decoy receptor IL-1R2 does not result in stimulation of a signaling cascade, as this receptor lacks a cytoplasmic segment **(C)**.

Both IL-1α and IL-1β signal by binding to the type 1 IL-1 receptor (IL-1R1). The family of IL-1 receptors also includes decoy receptors^15^ (such as IL-1R2) that do not trigger signaling, but serve as molecular sinks for the cytokine, terminating the IL-1-driven response. Regulation of IL-1 signaling is also modulated by endogenous natural inhibitors, such as IL-1 receptor antagonist (IL-1Ra). IL-1Ra is an endogenous natural inhibitor that binds to IL-1R1 but does not activate signaling, preventing recruitment of the IL-1 receptor accessory protein (IL-1RAcP), an essential component of the IL-1 receptor signaling system (**Figure 1**)^16^.

## 3. IL-1 upregulation in myocardial infarction

IL-1α release and IL-1β induction are consistently noted in experimental models of myocardial infarction^17,18^. Necrotic cardiomyocytes release IL-1α^18^, whereas pro-inflammatory monocyte subsets may be a major source of IL-1β in the infarcted heart^19^. Increased levels of IL-1 in human patients with myocardial infarction have been less consistently documented. In patients with ST elevation myocardial infarction (STEMI), circulating IL-1β levels were associated with systolic dysfunction and adverse remodeling^20^. However, other studies did not demonstrate elevated IL-1β levels in patients with myocardial infarction^21^. Such findings may reflect difficulties in detecting plasma IL-1β, due to binding of the cytokine to large proteins such as a2 macroglobulins, complement, and soluble receptors^22,23^. Activation of the inflammasome has been documented in many cell types involved in cardiac repair and is critically involved in generation of bioactive IL-1. Activation of the inflammasome in cardiac fibroblasts and in cardiomyocytes has been extensively documented in experimental models of myocardial infarction and may contribute to the post-infarction inflammatory reaction extending injury^24-26^.

Several other members of the IL-1 family are overexpressed in the infarcted heart. IL-18 is upregulated in the infarcted myocardium^27,28^; circulating IL-18 levels are increased in patients with acute coronary syndromes^29^. IL-33 is a biomechanically induced protein^30^ that is primarily expressed in fibroblasts and may be also upregulated following myocardial infarction^31^. IL-33 signals through binding to the orphan receptor ST2, increased levels of soluble ST2 have been associated with adverse outcome in patients with STEMI^32^. The endogenous receptor antagonist IL-1Ra is also upregulated in experimental models of myocardial infarction and is localized in the infarct border zone^33^. In human patients with myocardial infarction, serum IL-1Ra levels are increased^34^, preceding the release of markers of necrosis^35^. Associative studies suggested that plasma IL-1Ra levels correlate with the extent of cardiomyocyte death^36^ and with the severity of hemodynamic compromise in patients with acute myocardial infarction^37^.

## **4. **The role of IL-1 in regulation of cardiac injury, repair and remodeling following myocardial infarction

A large body of evidence, derived from genetic loss-of-function studies and antibody neutralization experiments suggests that members of the IL-1 family play crucial roles in injury, repair and remodeling of the infarcted heart^38-40^. The effects of IL-1 family members may involve several distinct actions on various cell types in the infarcted and remodeling heart.

### *4.1 * Does IL-1 extend ischemic injury following myocardial infarction?

A recent investigation demonstrated that IL-1α, released from necrotic cardiomyocytes, serves a crucial danger signal, implicated in activation of the post-infarction inflammatory response^18^. It has been suggested that release of constitutive IL-1α and induction of IL-1β may extend ischemic injury, increasing apoptosis of cardiomyocytes. *In vitro* experiments have demonstrated that IL-1β stimulation activates apoptotic pathways in neonatal rat cardiomyocytes^41^. Moreover, incubation of rat cardiomyocytes with recombinant human IL-1Ra (anakinra) reduced apoptosis in a simulated ischemia/reperfusion protocol. *In vivo*, overexpression of human IL-1Ra through gene transfection in heterotopically transplanted rat hearts undergoing ischemia and reperfusion significantly attenuated infarct size, reducing the number of apoptotic cardiomyocytes^42^. Pro-apoptotic effects of IL-1 were further supported by studies in rodent models of infarction showing that administration of recombinant human IL-1Ra decreased cardiomyocyte apoptosis and prevented cardiac dilation^43^. It should be noted that not all investigations suggested effects of IL-1 on the size of the infarct. IL-1R1 loss had no effect on the size of the infarct in a model of myocardial ischemia/reperfusion despite a marked attenuation in the inflammatory response^44^.

### *4.2 * IL-1 signaling is critically involved in activation of the post-infarction inflammatory response

**Figure 2:**
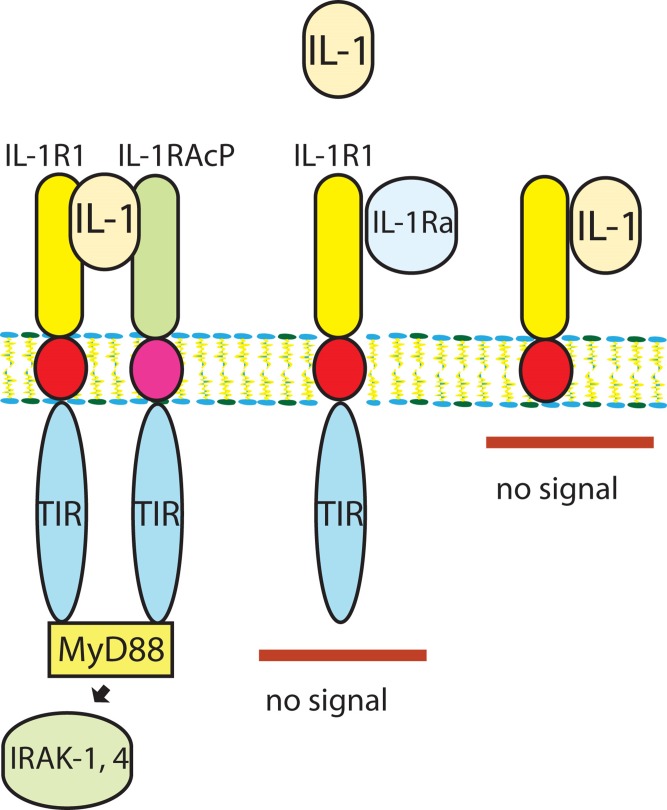
Figure 2. The cellular targets of IL-1 in myocardial infarction. IL-1α (released by necrotic cardiomyocytes) and IL-1β (newly synthesized and secreted by resident myocardial cells and infiltrating leukocytes) signal by activating IL-1R1. IL-1 induces cardiomyocyte apoptosis and suppresses cardiomyocyte function, stimulates a matrix-degrading pro-inflammatory program in cardiac fibroblasts and delays fibroblast to myofibroblast transdifferentiation, induces cytokine expression in macrophages, mediates leukocyte recruitment by inducing adhesion molecule expression by endothelial cells and prolongs neutrophil survival.

The role of IL-1 in activation of the post-infarction inflammatory response is supported by extensive in vivo and in vitro experimentation. IL-1 activates a pro-inflammatory program in all cells involved in cardiac injury and repair (Figure 2). In endothelial cells, IL-1 induces chemokine and adhesion molecule synthesis, enhancing adhesive interactions implicated in recruitment of leukocytes in injured tissues^45^. IL-1 also upregulates chemokine synthesis in mononuclear cells and prolongs the lifespan of neutrophils^46^. In vivo, IL-1Ra overexpression significantly reduced infiltration of the ischemic heart with neutrophils^42^ and IL-1R1 loss was associated with a marked reduction of peak cytokine and chemokine mRNA expression in the infarcted heart and with attenuated infiltration of the infarct with neutrophils and pro-inflammatory monocytes^19,44^. Attenuated inflammation in the absence of IL-1 does not result from a reduction in the size of the infarct, but primarily reflects direct IL-1-mediated pro-inflammatory actions^19,44^.

### *4.3 * Effects of IL-1 on fibroblast activation and on extracellular matrix metabolism

During the inflammatory phase of cardiac repair, resident cardiac fibroblasts undergo pro-inflammatory activation^47^ and may serve as an important source of cytokines and chemokines. Release of Il-1α, induction of IL-1β and downstream activation of IL-1R1 signaling stimulate an inflammatory program in cardiac fibroblasts^18,19,48^. In addition to its pro-inflammatory actions, IL-1 also promotes a matrix-degrading phenotype in cardiac fibroblasts, markedly upregulating synthesis of matrix metalloproteinases (MMPs)^49,50^. Moreover, activation of IL-1 signaling delays myofibroblast transdifferentiation reducing expression of a-smooth muscle actin in cardiac fibroblasts^19^. Thus, IL-1 signaling may prevent premature conversion of cardiac fibroblasts into matrix-synthetic myofibroblasts, until the wound is cleared from dead cells and matrix debris.

### *4.4 * IL-1 promotes adverse dilative remodeling of the infarcted heart

IL-1Ra overexpression studies and loss-of-function experiments targeting the IL-1 signaling cascade demonstrated that disruption of IL-1 attenuates dilative remodeling following myocardial infarction^44,51^. The beneficial actions of IL-1 disruption in post-infarction remodeling may be mediated through attenuation of pro-inflammatory signaling, or through loss of direct IL-1-mediated actions on matrix metabolism and on function of cardiac fibroblasts. Excessive matrix degradation reduces the tensile strength of the wound and may deprive surviving cardiomyocytes in the border zone from key pro-survival signals^52,53^.

### *4.5 * Termination of IL-1 signaling

Repair of the infarcted heart is dependent on timely repression of the inflammatory reaction and subsequent resolution of the inflammatory infiltrate^1^. Suppression of the inflammatory reaction is not a passive process, but requires activation of STOP signals that inhibit pro-inflammatory signaling. Considering the intense pro-inflammatory actions of IL-1, suppression and termination of IL-1 signaling is crucial for the transition from inflammation to repair. Several molecular signals may participate in suppression of the IL-1 response. First, induction of secreted anti-inflammatory mediators, such as IL-10^54^ and Transforming Growth Factor (TGF)-β^55^, may deactivate mononuclear cells, reducing IL-1 transcription. Although this is a plausible hypothesis, it should be noted that IL-10 null animals and wildtype controls had comparable myocardial IL-1β mRNA levels 24 h after reperfused infarction^56^. Second, mediators inhibiting the inflammasome may limit generation of bioactive IL-1β^57^. Third, activation of intracellular negative regulators, such as Interleukin receptor-associated kinase (IRAK)-M in macrophages and fibroblasts suppresses pro-inflammatory IL-1/Toll Like Receptor (TLR) signaling^58^. Fourth, upregulation of decoy receptors (such as IL-1R2) in modulated M2 macrophages of the infarct may serve as a molecular sink that terminates the IL-1 response^19^. Recruitment of monocyte and lymphocyte subsets with inhibitory properties may play an important role in suppressing the IL-1 response^59-61^.

### *4.6 * Does IL-1 exert protective actions on the infarcted heart?

Cytokines are highly pleiotropic mediators, exerting a wide range of actions on all cell types implicated in cardiac repair. The recent experience with interventions targeting the Tumor Necrosis Factor (TNF)-a system in patients with heart failure highlighted the unpredictable consequences of interfering with cytokine signaling^62^. TNF-a exerts both protective and injurious actions on the failing heart^63,64^; as a result, anti-TNF therapy did not prove beneficial in heart failure patients. Although the bulk of experimental evidence indicates that activation of IL-1 signaling exerts deleterious effects on the infarcted and remodeling heart, could IL-1 activation regulate pathways with a critical role in cardioprotection, or in repair of the injured myocardium?

Hard evidence suggesting protective effects of IL-1β on ischemic cardiomyocytes is lacking. However, a study using antibody neutralization to neutralize IL-1β suggested important effects of IL-1 in cardiac repair. Administration of a single intraperitoneal injection of a neutralizing anti-IL-1β antibody immediately after coronary ligation in a model of non-reperfused infarction significantly increased the incidence of cardiac rupture, reducing collagen accumulation in the infarcted area. Surviving animals exhibited accentuated chamber dilation^65^. The findings of this study are in conflict with several other investigations that showed attenuated adverse remodeling in animals with defective IL-1 signaling^44^ and in animals treated with IL-1 antagonists^42,43,51^. The basis for the contradictory findings is unclear. The differences in outcome may be related, at least in part, to the use of non-reperfused vs. reperfused models of myocardial infarction. Reperfused infarction is associated with more intense and early activation of pro-inflammatory signaling cascades. Moreover, in the presence of a permanently occluded coronary, early administration of an anti-IL-1 antibody will likely exclusively target the perfused non-infarcted area and may not significantly affect the reparative response in the infarct. Effectiveness of IL-1 inhibition may also depend on the dose and method used for IL-1 neutralization. Timing of the therapeutic intervention may also play a critical role in determining outcome.

## **5. **IL-1 as a therapeutic target in human patients with acute myocardial infarction

A growing body of experimental evidence suggests an important role for IL-1-driven inflammation in the pathogenesis of atherothrombotic disease. The availability of anakinra, a nonglycosylated recombinant human IL-1Ra that binds to IL-1R1 competitively inhibiting IL-1 signaling, provides an interesting and safe tool for therapeutic intervention. Anakinra has been approved for treatment of patients with rheumatoid arthritis with poor responses to disease modifying agents. Clinical trials examining the effects of IL-1β antibody inhibition on the incidence of cardiovascular events in high-risk patients are currently underway^66^. Considering the evidence suggesting a role for IL-1 in post-infarction remodeling, acute myocardial infarction represents a promising opportunity for the therapeutic use of IL-1 antagonists. The effectiveness of IL-1 blockade in patients with myocardial infarction was studied in small clinical investigations. Pilot studies suggested that in patients with STEMI, a 2-week course of anakinra is safe, may attenuate adverse remodeling^67^ and may reduce the incidence of post-infarction heart failure^68,69^. Large clinical studies are needed to test the effectiveness of IL-1 inhibition in STEMI patients. It should be emphasized that anti-IL-1 approaches may be particularly beneficial in patient subpopulations with overactive and prolonged post-infarction inflammatory responses^70^. These patients could be identified through the use of carefully selected biomarkers or imaging strategies^71^.

## **6. **Other members of the IL-1 family: the role of IL-18 and IL-33

Although IL-1α and IL-1β remain the best-studied members of the family, emerging evidence implicates IL-18 and IL-33 in regulation of the post-infarction inflammatory reaction. IL-18 inhibition studies have added IL-18 to the long list of pro-inflammatory mediators that may extend ischemic injury in mouse models^72^. Moreover, therapy with mesenchymal stem cells expressing IL-18 binding protein, a natural IL-18 inhibitor, protected the ischemic heart^73^. A role for IL-18 in mediating IL-1-dependent pro-inflammatory actions has been suggested^28^.

IL-33, on the other hand, exerts a wide range of pro-inflammatory actions, activating dendritic cells and enhancing LPS-dependent cytokine synthesis by macrophages^12^. In the healing infarct, IL-33 has protective actions attenuating cardiomyocyte apoptosis through binding to ST2^31^. The mechanisms responsible for the cardioprotective effects of the IL-33/ST2 axis remain poorly understood.

## 7. Conclusions

Despite intensive efforts, implementation of strategies targeting the inflammatory cascade in patients with myocardial infarction has been unsuccessful. The pleiotropic effects of inflammatory mediators that exert both beneficial and detrimental actions on many different cell types pose major challenges in designing an effective treatment approach. Redundant actions of various inflammatory mediators further complicate translational efforts.

Considering the strong evidence suggesting a key role for IL-1 in myocardial injury and remodeling, and the availability of safe and effective agents for IL-1 inhibition, IL-1 blockade should be considered a highly promising therapeutic approach in myocardial infarction. Studies in rodent models suggest a crucial role for IL-1 in activation of the post-infarction inflammatory reaction. IL-1 signaling also regulates fibroblast phenotype inducing a matrix-degrading program and promoting dilative remodeling. Early clinical studies testing the effectiveness of anti-IL-1 approaches in patients with acute myocardial infarction have produced promising results. Future studies need to expand our understanding of the role of IL-1 family members in the pathophysiology of myocardial infarction, while aggressively pursuing clinical translation. The significance of specific cellular actions of IL-1 on cardiomyocytes, immune cells, fibroblasts and vascular cells needs to be dissected *in vivo*. The mechanisms of IL-1-mediated injury need to be studied; the possibility for cytoprotective actions should be carefully considered. The molecular signals involved in negative regulation of the IL-1 response in the healing infarct need to be understood. Pathophysiologic functions of the newer members of the IL-1 family need to be explored. Understanding the biology of the IL-1 system in myocardial infarction is essential in order to design therapies for attenuation of post-infarction remodeling and for protection from the development of heart failure.

## KEY POINTS

**◊** **Several members of the Interleukin (IL)-1 family, including IL-1a, IL-1b, IL-18, IL-33 and the endogenous inhibitor IL-1Ra are released in the infarcted myocardium.**

**◊** **IL-1α is released by necrotic cells, whereas IL-1β is synthesized and activated through a molecular platform called the “inflammasone”.**

**◊** **Both IL-1α and IL-1β signal through the type 1 IL-1 receptor (IL-1R1). IL-1R1 plays a critical role in activation of post-infarction inflammation, promotes a matrix-degrading phenotype in fibroblasts, delays myofibroblast transdifferentiation and may induce cardiomyocyte apoptosis.**

**◊** **IL-1 mediates adverse remodeling and dysfunction following myocardial infarction.**

**◊Pilot clinical studies suggest that IL-1 neutralization may attenuate adverse remodeling and heart failure in patients with acute myocardial infarction**.

## OPEN Question

**◊** **Which cellular effects of IL-1 are critical in the pathogenesis of post-infarction remodeling?**
